# *Notes from the Field:* Childhood Lead Poisoning Associated with Turmeric Spices — Las Vegas, 2019

**DOI:** 10.15585/mmwr.mm7045a4

**Published:** 2021-11-12

**Authors:** Matthew Kappel, Vit Kraushaar, Arthuro Mehretu, Westol Slater, Erika Marquez

**Affiliations:** ^1^Division of Community Health, Office of Epidemiology and Disease Surveillance, Southern Nevada Health District, Las Vegas, Nevada; ^2^Division of Environmental Health, Southern Nevada Health District, Las Vegas, Nevada; ^3^Department of Environmental and Occupational Health, School of Public Health, University of Nevada, Las Vegas.

In March 2019, the Office of Epidemiology and Disease Surveillance of the Southern Nevada Health District (SNHD) was contacted by a local pediatrician regarding a developmentally normal boy aged 2 years (child A) with a high venous blood lead level (BLL) of 48 *μ*g/dL (reference range <5 *μ*g/dL) obtained during a routine well-child visit.* The pediatrician was not aware of any obvious source of lead exposure and also reported that child A’s cousin, a girl aged 9 months (child B), who lived in a different household, also had a high venous BLL of 11 *μ*g/dL. The parents in both families had immigrated from Afghanistan; both children were born in the United States.

Child A was admitted to a local hospital for a 2-day inpatient evaluation and treatment. The Poison Control Center recommended oral chelation therapy with succimer; however, because no succimer was locally available at the time of evaluation, succimer therapy (10 mg per kg body weight twice daily for 14 days) was scheduled to be initiated shortly after hospital discharge and after the home had undergone a lead assessment.^†^ During hospitalization, child A’s hemoglobin was 12.3 g/dL (reference range 11.0–12.8 g/dL), with red blood cell microcytosis (mean corpuscular volume = 72.5 fL [reference range 76.8–83.3 fL]) and hypochromia (mean corpuscular hemoglobin concentration = 32.3 g/dL [reference range 34.2–35.7 g/dL]). Ferritin level was 8 ng/mL (reference range = 7–140 ng/mL).

A standardized questionnaire administered to both families by SNHD did not initially identify potential sources of lead exposure. Child A’s parents live in an apartment built in 2013. Child B’s parents live in a single-family home built in 2005. No occupational exposures were identified. A certified assessor conducted a lead-risk assessment to identify and recommend removal of lead sources in both homes. Painted and nonpainted surfaces were tested using calibrated Niton XL3t-700 and calibrated Niton XL3p-303A-ray fluorescence (XRF) analyzers.

In child A’s home, several pieces of crockery, a meat grinder, turmeric spice, and a rice seasoning spice were identified as lead hazards by XRF. The turmeric and rice seasoning spices were purchased from a local market, and samples collected from the home were sent to an environmental laboratory accredited by the National Lead Laboratory Accreditation Program (NLLAP), which confirmed lead levels of 2,000 mg/kg (turmeric) and 0.6 mg/kg (rice seasoning) by atomic absorption spectroscopy (AAS).

In child B’s home, lead hazards identified by XRF included several pieces of crockery, floor tile, and two types of turmeric spice, one imported from Afghanistan and the other from the same local market as that of the turmeric found in child A’s home. Dust from the floor tile had an average lead level of 0.50 *μ*g/ft^2^ (Environmental Protection Agency clearance level = 10 *μ*g/ft^2^).^§^ AAS testing found lead levels of 15,000 mg/kg and 3,000 mg/kg in the turmeric from Afghanistan and from the local market, respectively.

The local acquisition of some of the leaded products raised concerns about potential continued exposure among vulnerable populations. Therefore, additional samples from the local market were obtained and tested; lead was not detected by XRF or by AAS analysis conducted by the NLLAP-accredited laboratory. Because the turmeric spice purchased from the local market had been removed from its original packaging, information regarding the product origin and lot number were not available. Child B’s family acknowledged purchasing the turmeric spice from the local market several months earlier.

Additional family members of both children were screened. Child A’s father had a venous BLL of 49 *μ*g/dL, and child B’s sister (aged 2 years) had a venous BLL of 13 *μ*g/dL. Interviews with both families indicated that the family of child A reportedly consumed larger quantities of turmeric-containing food than did the family of child B. Both families were advised to discontinue use of the lead-containing turmeric, obtain turmeric from reputable brands, and were provided nutritional counseling stressing the importance of a diet consisting of foods rich in calcium, iron, and vitamin C. Child A’s BLL was 18 *μ*g/dL in April 2019 after initiation of chelation therapy and was 9 *μ*g/dL by December. Repeat testing of BLLs in child B and her sister found that their BLLs had both declined to 3 *μ*g/dL by September.

These findings support other reports of lead-contaminated turmeric in the United States (*1*,*2*) and highlight the diverse pathways through which children can be exposed to lead. They underscore the importance of a multidisciplinary approach and communication between health care providers and health department staff members in identifying potential links among lead poisoning cases, and the need for health care facilities to be prepared to respond to cases of lead poisoning.

The national blood lead reference level had been 5 *μ*g/dL but was lowered to 3.5 *μ*g/dL in October 2021 (*3*). There is no safe BLL in children (*4*); BLLs once thought to pose little to no risk have shown to be risk factors for reading problems, intellectual delays, school failure, attention deficit-hyperactivity disorder, and antisocial behavior (*3**,**5*–*7*). Whereas the impact of lead exposure might be irreversible, exposure is preventable.^¶^^,^** Clinicians and public health professionals should be aware of risks outside traditional lead exposures (e.g., paint, dust, and contaminated soil). Adulteration of turmeric has reportedly been a source of lead exposure in other countries (*1*), where lead is purposefully added to enhance weight and color (*2*). Referrals for lead-risk assessments should emphasize same-day assessments when possible to reduce continued exposure to and absorption of lead. Public health officials and health care providers should work together to ensure the sources of lead exposure have been identified and controlled before chelation therapy is started. Health care providers who are unfamiliar with chelation therapy should consult with their regional pediatric environmental health specialty unit or poison control center for assistance.

## Footnotes

* At the time of publication, the CDC blood lead level reference (BLRV) is ≥3.5 *μ*g/dL. The BLRV is based on the 97.5th percentile of the National Health and Nutrition Examination Survey blood lead distribution in children aged 1–5 years. The BLRV is used to identify children with BLLs that are much higher than most children’s BLLs. https://www.cdc.gov/nceh/lead/data/blood-lead-reference-value.htm

† Hospital records did not indicate whether any other chelating agents were considered or available.

§ https://www.epa.gov/lead/hazard-standards-and-clearance-levels-lead-paint-dust-and-soil-tsca-sections-402-and-403 (Accessed January 23, 2020).

¶ https://www.who.int/news-room/fact-sheets/detail/lead-poisoning-and-health (Accessed January 23, 2020).

** https://www.atsdr.cdc.gov/csem/leadtoxicity/physiological_effects.html (Accessed January 23, 2020).
